# Supplementary material for: Learning curve and surgical outcome of robotic assisted colorectal surgery with ERAS program
Source: Sci Rep. 2022 Nov 29;12:20566. doi: 10.1038/s41598-022-24665-w (PMC9709162; doi:10.1038/s41598-022-24665-w)
Supplement: Supplementary file 1 — Supplementary Information 1. [file 41598_2022_24665_MOESM1_ESM.zip › LSC Raw data-Ñ[▒K/059/HS_ñw╝╨░O▒Kñσ.pdf]

正式摘要(電子簽章：0000001844533682)		
W82 / W82 ☐ 英文標題(注意:無英文姓名-請補病人基本檔!)	☐【讀取】憑證	

臺中榮民總醫院

出院病歷摘要

轉入號： 轉出號：

[redacted]

(8)流水編號	
(9)入院日期	109/01/08 (2020) 直腸外科 W82 - 112
(10)轉科日期	109/01/17 (2020) - 腎臟科
(11)出院日期	109/01/24 (2020) 住院天數計 16 日

診斷

- 入院：1. Rectal cancer, adenocarcinoma dignosed under colonscope biopsy on 108/12/31
  [Past History]
  1. ESRD, unknown cause, s/p renal transplantation on 2005/9/20 in China; now CKD, stage 4 (baseline Cr: 2.0~2.5), under Prograf (1 mg Q12H) and Cellcept (500 mg BID)
  2. Prostate cancer, cT1c, Gleason score 3+3=6, PSA: 28.71 (2017/3/1), s/p laparoscopic radical prostatectomy on 2017/5/8 (PSA on 2018/7/30: 0.008)
  3. Atrial fibrillation, CHA2DS2-VASc: 2
  4. BPH, under Tadalafil
  5. GU, Forrest III (EGD on 2018/11/14); now under PPI
  6. Veno-occlusive disease of bilateral forearm (total occlusion of right subclavian vein and right brachiocephalic vein), complicated with intermittent cellulitis
  7. Hemorrhoids, s/p intervention x3
  8. Gout
  9. Suspected hepatic hemangioma at S6-S7
  10. Perianal abscess, left semi house shoe type, s/p fistulotomy on 2019/12/23, keep QID 沖洗, s/p Oxacillin 219/12/24-
- 出院：1. Rectal cancer, adenocarcinoma, cT3N0M0, 10cm FAV s/p LAR on 2020/01/09
  2. Pneumonia s/p Tazocin(2020/1/16-1/23)
  3. Veno-occlusive disease of bilateral forearm with SVC syndrome
  4. ESRD, unknown cause, s/p renal transplantation on 2005/9/20 in China; now CKD, stage 4 (baseline Cr: 2.0~2.5), under Prograf (1 mg Q12H) and prednisolon
  [Past History]
  1. Prostate cancer, cT1c, Gleason score 3+3=6, PSA: 28.71 (2017/3/1), s/p laparoscopic radical prostatectomy on 2017/5/8 (PSA on 2018/7/30: 0.008)
  2. Atrial fibrillation, CHA2DS2-VASc: 2
  3. BPH, under Tadalafil
  4. GU, Forrest III (EGD on 2018/11/14); now under PPI
  5. Veno-occlusive disease of bilateral forearm (total occlusion of right subclavian vein and right brachiocephalic vein), complicated with intermittent cellulitis
  6. Hemorrhoids, s/p intervention x3
  7. Gout
  8. Suspected hepatic hemangioma at S6-S7
  9. Perianal abscess, left semi house shoe type, s/p fistulotomy on 2019/12/23, keep QID 沖洗, s/p Oxacillin 219/12/24-

主訴

- incidentaly findings or rectal ca by CT

病史

- This 81-year-old male had underlying diseases of:
  1. ESRD, unknown cause, s/p renal transplantation on 2005/9/20 in China; now CKD, stage 4 (baseline Cr: 2.0~2.5), under Prograf (1 mg Q12H) and Cellcept (500 mg BID)
  2. Prostate cancer, cT1c, Gleason score 3+3=6, PSA: 28.71 (2017/3/1), s/p laparoscopic radical prostatectomy on 2017/5/8 (PSA on 2018/7/30: 0.008)
  3. Atrial fibrillation, CHA2DS2-VASc: 2

- 4. BPH, under Tadalafil
5. GU, Forrest III (EGD on 2018/11/14); now under PPI
6. Veno-occlusive disease of bilateral forearm (total occlusion of right subclavian vein and right brachiocephalic vein), complicated with intermittent cellulitis
7. Hemorrhoids, s/p intervention x3
8. Gout
9. Suspected hepatic hemangioma at S6-S7
He was a government employee in the past. He had habits of social smoking and social drinking and had been quit since 62 years old. He lived with his wife and two daughters. And his ADL was partial dependent since last year

Last admission, the patient had perianal abscess and recieved abdominal CT scan for survey. Which incidentally found rectal cancer FAV 10cm. s/p Biopsy confirmed adenocarcinoma. This time, he was admitted for schedule laparoscopic low anterior resection and ileostomy.

體檢發現

- Cr=1.06-1.12-1.1
GOT/GPT=24/23
urine:Reactive urothelial cells present
jaundice
PCR: 0.15
Alb/Cr: 37.5
preformed Ab: class I (-), class II (-)
still cyanosis of right land and edema (+)
CA-199:53.67
    - General appearance :
        - easy looking , conscious clear , without cardiopulmonary distress
        - Vital signs自動帶入date 體溫(BT) 脈搏(P) 呼吸(R) 血壓(BP) 身高(BH) 體重(BW) BMI
20200108 36.6℃ 67/min 19次/分 126/64mmHg 154.3cm 64.7kg 27.2
    - HEENT :
        - Eyes: no anemic conjunctiva, no icteric sclera, normal EOM
        - ENT: no otorrhea, no rhinorrhea, no erythematous mucosa, no injected tonsil, no deformity
    - Neck: supple, freely movable, trachea midline,no deformity, no engorged jugular vein , no palpable thyroid or lymph node enlargement
    - Chest:
        - Inspection: Grossly normal, symmetric and fully expansion, no accessory muscle used, no operation scar, no spider angiomata, no gynecomastia
        - Palpation: no palpable mass or nodule, no subcutaneous emphysema, no tenderness
        - Ausculation: clear breathing sound
    - Heart:
        - Percussion: normal heart size
        - Ausculation: regular heart beat, normal S1, S2, no gallop, no murmur
    - Abdomen:
        - Inspection: in
            - flat contour, symetrically, no scar, no stria, no dilated veins, no rashes
        - Ausculation: normoactive bowel sound, no abdominal bruits
        - palpation: soft, no muscle guarding or rigidity, no local tenderness, no rebounding pain, no palpable mass, no palpable pulsation
        - Percussion: no shifting dulllness, no hepatomegaly, no splenomagaly
    -

Peripheral pulsation :	CA	BA	RA	FA	PA	PTA	DPA
Right side :	++	++	++	++	++	++	++/+++
Left side :	++	++	++	++	++	++	++/+++

    - Genito-anus: no tenderness or palpable mass lesion or rectal shelf, normal external genitalia, normal anal tone, brown stool

手術日期及方法

- 手術日期及方法 :20200109 1. Laparoscopic low anterior resection, end-to-end anastomosis, EC60, SDH 33mm.

- Bilateral doughnut: intact, Air leak test: done, no air leak.
  2. Loop ileostomy at RLQ.
  3. High ligation (+) above the left colic artery.
  4. Cut margin: proximal 9cm, distal 2cm.
  5. Take down of splenic flexure: nil.
  6. A 12mm hemovac placed in the pelvis.

住院治療經過

- After admission, he received laparoscopic low anterior resection with anastomosis and protection loop ileostomy on 2020/01/09. He tolerated the surgery well and recovered as schedule. All stiches and drainage tube are removed on 2020/01/17. However, the patient suffered from shortness of breath and much sticky sputum since 2020/01/15. We applied chest care and the usage of prophylatic antibiotics of Tazocin. His chest x-ray showed no pneumonia patch nor pulmonary edema. We also found the patient's bilateral upper limbs swelling, especially right upper limb. His both hands were cyanosis and numbness, so we prescribed promostan and consulted CV doctor. However, due to past history of DVT of right upper limb and highly suspicious CTO of junction of SVC and right subclavian vein, PTA will cause high risk of kidney injury. The patient and his family hesitated. Due to his histroy of kidney tranplantation and the usage of immunosuprresion agents, CRS doctor contact NEPH doctor for further medical care advice. After the discussion, the patient was transferred to NEPH duty. After transferred to NEPH, we prescribed lasix with albumin to keep I/O negative due to suspect SVC syndrome that caused him shortness of breath and wheezing breathing sound. After treatment, vital signs became stable and his respiratory pattern became smooth. Follow-up creatinine level was mild elevated, so we discontinued metolazone, spironolactone to keep I/O balnce. The patient could gradually stand up with other's support. Further lab data showed improving renal function. He may discharge and follow up in OPD.

合併症

一般檢查

DATE	TIME	ALB	TP	BIL.T	BIL.D	ALKP	AST	ALT	LDH	CHOL	TG	HDL-C	LDL	UA	NH3	AMY
1081212	1602			1.11		88		11								
1081213	0926	3.5														
1081214	1420			0.57					260							29
1081214	2054															24
1081215	1442	2.8														
1081215	2046			1.1	0.5											
1081218	1548			1.24	0.45											
1081220	0850	4.0		1.20	0.39	105	34	27	212							
1081223	0903			1.00												
1081224	1713	3.4		0.8		90	20	17	180							66
1090107	1314	3.5		0.43	0.19	130	36	21	154							
1090109	1812	3.3		0.4	0.2	115	20	14								
1090113	1051	3.1		0.43			14	3								
1090116	0902			0.37	0.16		19	3								
1090116	0902						19	3								
1090116	0903														110	
1090117	0919			0.50				3								
1090118	1503														92	
1090118	2130	3.3		0.89				4	172							
1090120	0904														68	
1090120	0951	3.2		0.79				4	208							
1090123	0929			0.76				10								

DATE	TIME	WBC	RBC	HGB	HCT	MCV	PLT	BLST	NEUT	BAND	SEG	LYM	MON	EOS	BAS
1081212	1555	27060		8.6			239			1	89	2	8		
1081214	1555	20210	3.22	7.6	24.6	76.4	205			1	86	3	10		
1081215	0902	10700	2.90	6.9			147		88.0			4.2	6.9	0.7	0.2
1081215	2045	12470	3.48	8.5	27.0	77.6	164		87.0			5.1	6.5	1.2	0.2
1081218	1552	7700	3.20	7.9	25.9	80.9	168		79.7			13.0	5.3	1.7	0.3
1081220	0859	8310		8.9			176		84.1			10.8	4.2	0.5	0.4
1081224	1716	8440		8.4			200		72.3			19.8	6.0	1.4	0.5
1081230	0951			8.6			260								
1090107	1314	6040	3.60	9.2	29.5	81.9	269		64.2			26.7	7.8	1.0	0.3
1090109	1811	9050		8.6	28.2		207		91.1			7.3	1.5	0.0	0.1
1090113	1046	10180	3.60	9.4	31.1	86.4	148		83.3			12.1	4.5	0.0	0.1
1090116	0918	8570	3.19	8.2	26.6	83.4	96		84.1			10.6	5.3	0.0	0.0
1090117	0854	8620		8.3	28.0		92		84.7			10.2	5.1	0.0	0.0

1090118	2131	9030	3.79	9.9			119		77.7			15.9	6.2	0.1	0.1
1090120	0934	13110	3.63	9.4	29.3	80.7	158		86.6			9.6	3.6	0.0	0.2
1090123	0847	9510	3.59	9.3	30.1	83.8	199		82.4			13.8	3.4	0.2	0.2

DATE	TIME	NA	K	CL	CA	BUN	CREAT	Mg	P	CRP	ESR	PCT	LIPASE	LACTATE
1081212	1601													25.9
1081212	1602	129	1.3			37	3.33			18.464				
1081212	1720		1.2					1.6						
1081213	0821													10.9
1081213	0926	133	1.4		8.3	43	3.61	2.2	3.1					
1081213	1610		1.5											
1081214	0859		1.6					2.3						
1081214	1420	144	2.4	111	8.1	43						2.470	30	
1081214	1555													8.1
1081214	2054												24	9.5
1081215	0802													10.4
1081215	0903		2.6											
1081215	0903		2.6											
1081215	1442		3.1											
1081215	2046	152	3.1				2.88	2.3	2.2					9.9
1081215	2047									12.436				
1081216	0633	155	3.4				2.83							
1081217	0538		3.1											
1081218	1548	162	3.7				2.30							
1081219	0610	163	3.7											
1081220	0850	162	4.5		8.7	27	2.20	2.4	3.1					
1081222	0838	152	4.0				2.72							
1081223	0903	145	3.9			40	2.53							
1081224	1712											0.309		
1081224	1713	140	3.6		8.0	46	2.73	2.3					70	17.6
1081226	0921	133	4.3			45	2.50							
1081230	0926	129	4.6			37	2.00							
1090107	1314	128	4.3		8.2	18	1.44							
1090109	1812	139	4.0		8.3	16	1.36							
1090113	1051	138	3.8			29	1.45							
1090116	0902	144	2.3			27	1.45							
1090116	0903													7.2
1090117	0743													6.2
1090117	0919	149	2.6			25	1.61							
1090118	1523	151	2.9					1.7						
1090118	2130	154	2.7	124	8.6	22	1.72	2.6	1.4			0.453		13.1
1090119	0847	151	2.8	123	8.5	27	1.88							
1090120	0951	151	2.7	122	8.7	37	2.10	2.6	2.0					
1090123	0929	146	3.0		9.0	37	2.00							

DATE	TIME	GLUCOSE	PAB	TRF	ZN	CU	KETONE	CK	CKMB	PT-P	PT-C	APTT-P	APTT-C	TRO-T
1081212	1556									12.8	10.1	40.5		
1081212	1602	154												
1081214	2052									12.4	10.1	37.1		
1081214	2054							183	3					66.88
1081215	0903							91	3					
1081215	0906													54.91
1081215	2046	132												
1081224	1709									13.4	10.1	32.2		
1081224	1713							70	6					57.04
1081230	0912									12.1	10.1	36.3		
1090107	1314	110								11.9	10.1	36.7		
1090118	2128									13.0	10.1	39.0		

DATE	TIME	SP-GR	PH	PROT	GLUC	KETON	BILI	UROB	OB	NITR	LEU	RBC	WBC	EPCEL
1081213	0541	1.013	6.0	1+	Negative	Negative	Negative	0.2	3+	Negative	Negative	10-20	2-5	0-1
1081214	1418	1.013	6.5	2+	Negative	Negative	Negative	0.2	3+	Negative	Negative	10-20	0-2	0-1
1081222	1532	1.010	7.5	1+	Negative	Negative	Negative	0.2	2+	Negative	Negative	0-2	0-2	0-1

DATE	TIME	STOOL-OB	FAT	RBC	WBC	PARA
1081214	1714	4+				
1081215	1512	1+		0 - 2	0 - 2	Not Found
1081215	2053			0 - 2	0 - 2	Not Found

DATE	TIME	PH	PCO2	PO2	SO2	BEB	HCO3	TCO2	HGB	FIO2

1081213	1029	7.205	47.7	282.4	99.8	-9.5	18.5	19.9		
1081214	1556	7.288	43.0	73.6	92.0	-6.3	20.1	21.4		
1081214	2053	7.332	35.6	250.0	99.8	-6.6	18.4	19.5		
1081215	0801	7.314	41.3	40.8	69.7	-5.4	20.5	21.8		
1081215	2047	7.365	35.0	221.9	99.7	-5.0	19.5	20.6		
1081218	1551	7.464	42.8	173.9	99.6	5.6	30.0	31.3		
1090116	0903	7.306	22.6	142.6	98.7	-13.8	11.0	11.7		
1090117	0743	7.329	24.0	155.9	99.1	-12.3	12.4	13.1		

DATE	TIME	PH	BEB	SCVO2	PO2	HCO3	HGB	PCO2	TCO2
1081212	1554	7.227	-8.0	63.6	41.1	19.7		48.4	21.2
1081213	0834	7.191	-9.4	89.0	73.2	19.1		51.0	20.6
1081220	0759	7.431	7.8	86.3	48.9	33.8		52.0	35.4
1081224	1705	7.452	5.4	72.5	35.6	30.1		44.1	31.4
1081226	0909	7.430	5.7	85.3	47.9	31.1		48.0	32.6
1090118	2129	7.336	-10.3	74.1	43.6	14.3		27.4	15.1
1090120	1053	7.398	-9.9	99.4	164.4	13.5		22.4	14.2
1090123	0845	7.407	-3.2	86.0	51.4	21.0		34.1	22.0

特殊檢查

病理報告

- 活體組織切片病理報告 手術日期：**109/01/09** 病理號：**S0901138**

診斷醫師:王任卿 報告日期:109/01/14
Pathologic diagnosis:
1. Rectum, lapa low anterior resection + ileostomy --- Adenocarcinoma, moderately differentiated, arising in a tubulovillous adenoma. Tumor invaded to serosa and metastasized to perirectal lymph node (1/18). Focal mucin pooling noted. The bilateral cut ends are free of tumor.
2. IMA root, dissection --- Unremarkable fibrovascular tissue and lymph node (0/1).
3. Rectum, cut end, distal, resection --- Chronic inflammation.

Ancillary study for diagnosis:
IHC stains for MLH1, MSH2, PMS2 and MSH6 are done on section E. All above markers are preserved.

Prognostic and predictive factor:
1. Tumor size: 5x3.5 cm.
2. Depth of invasion: Tumor invades to visceral peritoneum (including tumor continuous with serosal surface through area of inflammation).
3. Histologic grade: G2: Moderately differentiated.
4. Angiolymphatic invasion: Present.
5. Perineural invasion: Not identified.
6. Tumor deposits (discontinuous extramural extension): Present, specify number of deposits: two.
7. Surgical Margins: a) Proximal Margin: Uninvolved by invasive carcinoma, distance of tumor from margin: 9 cm. b) Distal Margin: Uninvolved by invasive carcinoma, distance of tumor from margin: 2.5 cm. c) Radial or Mesenteric Margin: Uninvolved by invasive carcinoma, distance of tumor from margin: 3 cm.
8. Lymph node status: Involved (1/19).
9. Extranodal involvement: Present.
10. Tumor regression grading S/P CCRT: No known presurgical therapy.
11. Type of polyp in which invasive carcinoma arose: Tubulovillous adenoma.
12. Additional pathologic findings: Not identified.
13. Pathological TNM Stage: pT4aN1a(According to the eighth edition, 2017, American Joint Committee on Cancer Staging Guidelines for Tumors / 2017 CAP guideline).
14. TNM descriptors: Not applicable.

Gross description:
The specimen consists of 1) a segment of colon, 13.5 cm in length and 4.5 cm in proximal cut end circumference, 6 cm in distal circumference, and perirectal soft tissue. A gray tan firm annular elevated and centrally ulcerative tumor, 5x3.5 cm, is noted, 2.5 cm

- to the distal cut end. On cross section, the tumor invades to the mesocolic soft tissue with 30 mm to the radial margin, and serosal retraction is also noted. The both cut ends are free of tumor grossly. 2) IMA root, a piece of tan yellow soft tissue, 3.2x2.5x0.6 cm. 3) distal cut end, a piece of tan mucosa-covered soft tissue, 1.6x1.5x0.5 cm. Representative part for section: A) proximal cut end B) distal cut end C-E) tumor F) colon G-H) specimen 2-3 J-L) lymph nodes.
  Note:
  1. The interpretation of MMR panel and recommended studies are as below: (1) No loss of nuclear expression of MMR proteins: low probability of microsatellite instability-high (MSI-H) . (2) Please note: There are exceptions to the above IHC interpretations. These results should not be considered in isolation, and clinical correlation with genetic counseling is recommended to assess the need for germline testing.
  2. This case has been peer reviewed by two doctors.
  #T-59600_2 #M-82633_2 2 1105 S0000F
  #T-C4520_2 #M-81406_2 2 1105 000000

其它

出院時情況

- 3.改門診治療

出院計劃

- 服藥內容:

藥名	劑量	劑量單位	用法	頻次	數量	數量單位	現況	天數	單位
(□)Rosis tab 40mg	1	TAB	PO	QDPRN	7	TAB	TH	7	天
Prednisolone tab 5mg	1	TAB	PO	QD	7	TAB	TH	7	天
Sodium bicarbonate tab 0.6G	1	TAB	PO	QD	7	TAB	TH	7	天

- 中文服藥用法及注意事項已列印於藥袋供病人參考
- 緊急連絡電話:04-23592525 轉分機6082

主治醫師_1162D 莊雅雯 / 住院醫師_1706K 林彥伯

正式摘要(電子簽章：000000184453682)

W82 / W82

doctorRank:B
